# Organization, evolution and functions of the human and mouse Ly6/uPAR family genes

**DOI:** 10.1186/s40246-016-0074-2

**Published:** 2016-04-21

**Authors:** Chelsea L. Loughner, Elspeth A. Bruford, Monica S. McAndrews, Emili E. Delp, Sudha Swamynathan, Shivalingappa K. Swamynathan

**Affiliations:** Department of Ophthalmology, University of Pittsburgh School of Medicine, Pittsburgh, USA; HUGO Gene Nomenclature Committee (HGNC), European Bioinformatics Institute (EMBL-EBI), Wellcome Genome Campus, Hinxton, Cambridgeshire CB10 1SD UK; Mouse Genome Informatics, The Jackson Laboratory, Bar Harbor, ME 04609 USA; Department of Cell Biology, University of Pittsburgh School of Medicine, Pittsburgh, USA; McGowan Institute of Regenerative Medicine, University of Pittsburgh, Pittsburgh, USA; Fox Center for Vision Restoration, University of Pittsburgh School of Medicine, Pittsburgh, USA; Eye and Ear Institute, University of Pittsburgh School of Medicine, 203 Lothrop Street, Room 1025, Pittsburgh, PA 15213 USA

**Keywords:** Ly6/uPAR family, LU domain, Three-finger domain, uPAR, Lymphocytes, Neutrophils

## Abstract

Members of the lymphocyte antigen-6 (Ly6)/urokinase-type plasminogen activator receptor (uPAR) superfamily of proteins are cysteine-rich proteins characterized by a distinct disulfide bridge pattern that creates the three-finger Ly6/uPAR (LU) domain. Although the Ly6/uPAR family proteins share a common structure, their expression patterns and functions vary. To date, 35 human and 61 mouse Ly6/uPAR family members have been identified. Based on their subcellular localization, these proteins are further classified as GPI-anchored on the cell membrane, or secreted. The genes encoding Ly6/uPAR family proteins are conserved across different species and are clustered in syntenic regions on human chromosomes 8, 19, 6 and 11, and mouse Chromosomes 15, 7, 17, and 9, respectively. Here, we review the human and mouse Ly6/uPAR family gene and protein structure and genomic organization, expression, functions, and evolution, and introduce new names for novel family members.

## Introduction

The lymphocyte antigen-6 (Ly6)/urokinase-type plasminogen activator receptor (uPAR) superfamily of structurally related proteins is characterized by the LU domain, an 80 amino acid domain containing ten cysteines arranged in a specific spacing pattern that allows distinct disulfide bridges which create the three-fingered (3F) structural motif [1, 2]. Ly6/uPAR proteins were first identified in the mouse over 35 years ago using antisera against lymphocytes [3]. Human homologs were subsequently isolated, leading to the recognition that they represent a well-conserved family with wide-ranging expression patterns and important functions. The fully annotated human and mouse genomes contain 35 and 61 Ly6/uPAR family members, respectively. Research over the last decade has begun to unravel the important functions of the encoded proteins. In this review, we provide an overview of the Ly6/uPAR gene family and their genomic organization, evolution, as well as functions, and provide a nomenclature system for the newly identified members of this family.

## Inclusion and approved nomenclature for novel Ly6/uPAR family members

Although Ly6/uPAR family members are related by their structure, absence of a uniform naming convention resulted in arbitrary nomenclature for these genes as they were discovered. As many of the currently approved gene symbols for Ly6/uPAR family members (e.g., *CD59* and *PLAUR*) have been widely used in the scientific literature for many years, we have refrained from a family-wide attempt to standardize their well-established names, avoiding the potential for additional confusion. In compiling this update, we came across many novel members of the Ly6/uPAR gene family, especially in the mouse genome, that did not yet have a systematic name. We named these novel family members in line with the Ly6/uPAR genes that they are most related to, based on a phylogenetic analysis (see below) using either the established LY6/Ly6# root for those that fell within the LY6 clades, or the LYPD/Lypd# (LY6/PLAUR domain-containing) root for those outside the LY6 clades. The new symbols for these genes (1 human and 18 mouse), approved by the HGNC (HUGO Gene Nomenclature Committee) [4, 5] and MGNC (Mouse Genomic Nomenclature Committee) [6], are listed in Tables 1 and 2, respectively. We use the newly approved names for these genes in the rest of this update. HGNC have also created a gene family web-page for the human Ly6/uPAR family members (http://www.genenames.org/cgi-bin/genefamilies/set/1226).

**Table 1:** Name, chromosomal location, number of exons, and LU domains for human Ly6/uPAR family genes

Approved gene symbol (NCBI accession #)	Gene name	Alias(es)	Genomic location	Number of	
				Exons	LU Domains
*ACRV1* (NP_001603.1)	Acrosomal vesicle protein 1	*SP-10; SPACA2; D11S4365*	11q24.2	5	1
*CD177* (BAE93254.1)	CD177 molecule (Human Neutrophil Alloantigen 2A)	*NB1; PRV1; HNA2A; PRV-1; HNA-2a; NB1 GP*	19q13.2	9	2
*CD59* (CAG46523.1)	CD59 molecule, complement regulatory protein	*1 F5; EJ16; EJ30; EL32; G344; MIN1; MIN2; MIN3; MIRL; HRF20; MACIF; MEM43; MIC11; MSK21; 16.3A5; HRF-20; MAC-IP; p18-20*	11p13	5	1
*GML* (EAW82296.1)	Glycosylphosphatidylinositol anchored molecule like	*LY6DL*	8q24.3	5	1
*GPIHBP1* (AAH35810.2)	Glycosylphosphatidylinositol anchored high density lipoprotein-binding protein 1	*HYPL1D; GPI-HBP1*	8q24.3	4	1
*LY6D* (AAH31330.1)	Lymphocyte antigen 6 complex, locus D	*E48; Ly-6D*	8q24	3	1
*LY6E* (AAH03392.1)	Lymphocyte antigen 6 complex, locus E	*RIGE; SCA2; RIG-E; SCA-2; TSA-1*	8q24.3	4	1
*LY6G5B* (CAC85543.1)	Lymphocyte antigen 6 complex, locus G5B	*G5b; C6orf19*	6p21.3	3	1
*LY6G5C* (CAC85542.1)	Lymphocyte antigen 6 complex, locus G5C	*G5C; NG33; C6orf20; LY6G5CA; LY6G5CB*	6p21	3	1
*LY6G6C* (EAX03491.1)	Lymphocyte antigen 6 complex, locus G6C	*G6c; NG24; C6orf24*	6p21	3	1
*LY6G6D* (CAC85540.1)	Lymphocyte antigen 6 complex, locus G6D	*G6D; NG25; LY6-D; MEGT1; C6orf23*	6p21.3	3	1
*LY6G6F* (AAI37213.1)	Lymphocyte antigen 6 complex, locus G6F	*G6f; NG32; LY6G6D; C6orf21*	6p21.33	6	1
*LY6H* (BAA34115.1)	Lymphocyte antigen 6 complex, locus H	*NMLY6*	8q24.3	4	1
*LY6K* (AAI17145.1)	Lymphocyte antigen 6 complex, locus K	*CT97; ly-6 K; URLC10; HSJ001348*	8q24.3	3	1
*LY6L* ^a^ (XP_011544859.1)	Lymphocyte antigen 6 complex, locus L	*LOC101928108*	8q24.3	5	1
*LYNX1* (NP_803429.1)	Ly6/neurotoxin 1		8q24.3	4	1
*LYPD1* (EAX11675.1)	Ly6/PLAUR domain containing 1	*PHTS; LYPDC1; LYNX2*	2q21.2	3	1
*LYPD2* (EAW82307.1)	Ly6/PLAUR domain containing 2	*LYPDC2; UNQ430*	8q24.3	3	1
*LYPD3* (EAW57190.1)	Ly6/PLAUR domain containing 3	*C4.4A*	19q13.31	5	2
*LYPD4* (AAH34629.1)	Ly6/PLAUR domain containing 4	*Sperm Membrane Receptor*	19q13.2	5	1
*LYPD5* (EAW57232.1)	Ly6/PLAUR domain containing 5	*PRO4356*	19q13.31	5	1
*LYPD6* (AAH47013.1)	Ly6/PLAUR domain containing 6		2q23.2	5	1
*LYPD6B* (AAH18203.1)	Ly6/PLAUR domain containing 6B	*CT116; LYPD7*	2q23.2	7	1
*LYPD8* (NP_001278212.1)	Ly6/PLAUR domain containing 8		1q44	7	1
*PATE1* (AAI07045.1)	Prostate and testis expressed 1	*PATE*	11q24.2	5	1
*PATE2* (AAI44527.1)	Prostate and testis expressed 2	*PATE-M; UNQ3112; C11orf38*	11q24.2	6	1
*PATE3* (NP_001123355.3)	Prostate and testis expressed 3	*HEL-127; PATE-DJ*	11q24.2	3	1
*PATE4* (NP_001138346.1)	Prostate and testis expressed 4	*PATE-B*	11q24.2	3	1
*PINLYP* (NP_001180550.1)	Phospholipase A2 inhibitor and Ly6/PLAUR domain containing		19q13.31	6	1
*PLAUR* (CAG33233.1)	Plasminogen activator, urokinase receptor	*CD87; UPAR; URKR; U-PAR*	19q13	7	3
*PSCA* (AAH65183.1)	Prostate stem cell antigen	*PRO232*	8q24.2	3	1
*SLURP1* (AAT01436.1)	Secreted Ly6/PLAUR domain containing 1	*ARS; MDM; ANUP; ArsB; LY6LS*	8q24.3	3	1
*SLURP2* (NP_803253.1)	Secreted Ly6/PLAUR domain containing 2	*LYNX1, isoform B precursor*	8q24.3	4	1
*SPACA4* (AAQ88753.1)	Sperm acrosome associated 4	*SAMP14*	19q13.33	1	1
*TEX101* (EAW57189.1)	Testis expressed 101	*SGRG; CT131; GTPR867; NYD-SP8; PRO1884; SPATA44; TES101RP*	19q13.31	9	1

^a^Novel gene named in this study

**Table 2:** Name, chromosomal location, number of exons and LU domains for mouse Ly6/uPAR family genes

Approved gene symbol (NCBI Accession #)	Gene name	Alias(es)	Genomic location	Number of	
				Exons	LU domains
*Acrv1* (EDL25409.1)	Acrosomal vesicle protein 1	*Msa63; SP-10*	9 A4	4	1
*Cd177* (AAH27283.1)	CD177 antigen	*Pdp3; 1190003K14Rik*	7 A3	17	4
*Cd59a* (AAL04433.1)	CD59a antigen	*Cd59; AA987121; protectin*	2 54.53 cM	6	1
*Cd59b* (AAL04434.1)	CD59b antigen		2 E2	9	1
*Gml* (CAB57316.1)	Glycosylphosphatidylinositol anchored molecule like	*HemT3; EG625599*	15 D3	5	1
*Gml2* (AAI19338.1)	Glycosylphosphatidylinositol anchored molecule like 2	*HemT; Hemt1; 1700057K19Rik*	15 D3	7	1
*Gpihbp1* (NP_081006.1)	Glycosylphosphatidylinositol anchored high density lipoprotein-binding protein 1	*GPI-HBP1; 1110002J19Rik*	15 E1	4	1
*Ly6a* (AAH02070.1)	Lymphocyte antigen 6 complex, locus A	*TAP; Sca1; Sca-1; Ly-6A.2; Ly-6A/E; Ly-6E.1*	15 34.29 cM	4	1
*Ly6a2* ^a^ (XP_006543305.1)	Lymphocyte antigen 6 complex, locus A2	*Ly6A-2/E-1, I830127L07Rik*	15 D3	4	1
*Ly6c1* (AAH10764.1)	Lymphocyte antigen 6 complex, locus C1	*Ly6c; Ly-6C; Ly-6C1; AA682074; AA959465*	15 34.29 cM	6	1
*Ly6c2* (NP_001092687.1)	Lymphocyte antigen 6 complex, locus C2	*Ly-6C2; Ly-6C.2*	15 D3	4	1
*Ly6d* (EDL29445.1)	Lymphocyte antigen 6 complex, locus D	*Thb; Ly61; Ly-61*	15 D3	3	1
*Ly6e* (CAJ18452.1)	Lymphocyte antigen 6 complex, locus E	*Ly67; Tsa1; RIG-E; Sca-2; TSA-1*	15 D3	5	1
*Ly6f* (EDL29474.1)	Lymphocyte antigen 6 complex, locus F		15 D3	4	1
*Ly6g* (NP_001297367.1)	Lymphocyte antigen 6 complex, locus G	*Gr1; Gr-1; Ly-6G*	15 D3	4	1
*Ly6g2* ^a^(AAH25446.1)	Lymphocyte antigen 6 complex, locus G2	*BC025446*	15 D3	6	1
*Ly6g5b* (CAC85549.1)	Lymphocyte antigen 6 complex, locus G5B		17 B1	3	1
*Ly6g5c* (CAC85548.1)	Lymphocyte antigen 6 complex, locus G5C	*G5c; NG33*	17 B1	3	1
*Ly6g6c* (AAI16367.1)	Lymphocyte antigen 6 complex, locus G6C	*G6c; NG24; AU016360; 1110003M04Rik*	17 B1	3	1
*Ly6g6d* (CAC85545.1)	Lymphocyte antigen 6 complex, locus G6D	*G6d; G6f; NG25; NG32; MEGT1; A930024F17Rik*	17 B1	4	1
*Ly6g6e* (AAI38778.1)	Lymphocyte antigen 6 complex, locus G6E	*G6e; 2310011I02Rik*	17 B2	3	1
*Ly6g6f* (AAI72069.1)	Lymphocyte antigen 6 complex, locus G6F	*CJ068215*	17 B1	6	1
*Ly6g6g* ^a^(EDL29446.1)	Lymphocyte antigen 6 complex, locus G6G	*D730001G18Rik*	15 D3	3	1
*Ly6h* (AAH28758.1)	Lymphocyte antigen 6 complex, locus H		15 E1	6	1
*Ly6i* (AAI45088.1)	Lymphocyte antigen 6 complex, locus I	*Ly-6I; Ly-6 M; AI789751*	15 D3	6	1
*Ly6k* (AAH49723.1)	Lymphocyte antigen 6 complex, locus K	*mLy-6 K; 2410015A16Rik; 3110035B01Rik*	15	4	1
*Ly6l* ^a^ (XP_006521679.1)	Lymphocyte antigen 6 complex, locus L	*Gm20654*	15 34.37 cM	3	1
*Ly6m* ^a^(EDL29458.1)	Lymphocyte antigen 6 complex, locus M	*2010109I03Rik*	15 D3	3	1
*Lynx1* (AAF16899.1)	Ly6/neurotoxin 1	*AI838844*	15 D3	4	1
*Lypd1* (AAH58599.1)	Ly6/PLAUR domain containing 1	*Lynx2; Lypdc1; AI853408; 2700050C12Rik; C530008O16Rik*	1 E3	6	1
*Lypd2* (AAI32410.1)	Ly6/PLAUR domain containing 2	*VLL; Lypdc2; 0610005K03Rik*	15 E1	4	1
*Lypd3* (AAH16549.1)	Ly6/PLAUR domain containing 3	*C4.4a; 2310061G07Rik*	7 A3	5	2
*Lypd4* (AAH49744.1)	Ly6/PLAUR domain containing 4	*4933400F01Rik*	7 A3	5	1
*Lypd5* (AAI07188.1)	Ly6/PLAUR domain containing 5	*2210003I03Rik*	7 A3	5	1
*Lypd6* (AAH70462.1)	Ly6/PLAUR domain containing 6	*E130115E03Rik*	2 C1.1	7	1
*Lypd6b* (AAI26944.1)	Ly6/PLAUR domain containing 6B	*AW049525; 2310010M24Rik*	2 C1.1	14	1
*Lypd8* (NP_001077353.1)	Ly6/PLAUR domain containing 8	*2210415F13Rik*	11 B1.3	8	1
*Lypd9* ^a^(AAH48595.1)	Ly6/PLAUR domain containing 9	*4930504O13Rik* *Gm524*	11 B1.3	4	1
*Lypd10* ^a^(BC049730.1)	Ly6/PLAUR domain containing 10	*BC049730*	7 A3	8	1
*Lypd11* ^a^(NP_808261.1)	Ly6/PLAUR domain containing 11	*Gm4763,* *EG210155*	7 A3	9	1
*Pate1* (NP_001186882.1)	Prostate and testis expressed 1	*Pate*	9 A4	5	1
*Pate2* (NP_001028593.1)	Prostate and testis expressed 2	*Gm846; Pate-M; mANLP1*	9 A4	10	1
*Pate3* (NP_001161064.1)	Prostate and testis expressed 3	*Pate-dj*	9 A4	3	1
*Pate4* (AAI20767.1)	Prostate and testis expressed 4	*Svs7; Pate-B; 9530004K16Rik*	9 A4	4	1
*Pate5* ^a^ (NP_084139.1)	Prostate and testis expressed 5	*9230110F15Rik, Pate-A, mANLP3*	9 A4	3	1
*Pate6* ^a^ (NP_080869.1)	Prostate and testis expressed 6	*D730048I06Rik, Pate-C, mANLP2*	9 A4	3	1
*Pate7* ^a^ (NP_001161145.1)	Prostate and testis expressed 7	*Pate-E, Gm17727*	9 A4	3	1
*Pate8* ^a^ (NP_001161056.1)	Prostate and testis expressed 8	*Pate-G,* *Gm17689*	9 A4	3	1
*Pate9* ^a^ (NP_001028955.1)	Prostate and testis expressed 9	*Pate-H, EG434396, Gm5615*	9 A4	3	1
*Pate10* ^a^ (ACD81927.1)	Prostate and testis expressed 10	*Pate-N, Gm17677*	9 A4	3	1
*Pate11* ^a^ (ACD81928.1)	Prostate and testis expressed 11	*Pate-P, Gm9513*	9 A4	4	1
*Pate12* ^a^ (NP_001161058.1)	Prostate and testis expressed 12	*Pate-Q, EG639025, Gm7257*	9 A4	4	1
*Pate13* ^a^ (XP_006510783.1)	Prostate and testis expressed 13	*9230113P08Rik*	9 A4	3	1
*Pate14* ^a^ (NP_001028497.1)	Prostate and testis expressed 14	*A630095E13Rik, Gm191; Sslp1*	9 A4	4	1
*Pinlyp* (NP_001032220.1)	Phospholipase A2 inhibitor and Ly6/PLAUR domain containing	*2310033E01Rik*	7 A2-A3	6	1
*Plaur* (NP_035243.1)	Plasminogen activator, urokinase receptor	*Cd87; uPAR; u-PAR*	7 A3	11	3
*Psca* (EDL29439.1)	Prostate stem cell antigen	*2210408B04Rik*	15 D3	3	1
*Slurp1* (EDL29441.1)	Secreted Ly6/PLAUR domain containing 1	*ARS; ArsB; Slurp-1; AI415082; 1110021N19Rik*	15 D3	3	1
*Slurp2* ^a^ (AAI15612.1)	Secreted Ly6/PLAUR domain containing 2	*2300005B03Rik*	15 D3	3	1
*Spaca4* (AAH48608.1)	Sperm acrosome associated 4	*Samp14; AV043694; 1700008E09Rik*	7 B4	1	1
*Tex101* (AAH48475.1)	Testis expressed 101	*AI429076; TES101RP; 1700008H15Rik*	7 A3	6	1

^a^Novel genes named in this study

## Genomic organization of the Ly6/uPAR gene family

The Ly6/uPAR gene family currently includes 35 well-characterized human members, while the mouse gene family is considerably larger with 61 genes. Information including the name, chromosomal location, numbers of exons and LU domains for human and mouse family members is summarized in Tables 1 and 2, respectively. Twelve human Ly6 genes are clustered together within a short span of about 500 kb on chromosome 8 (8q24) (moving outward from the center of the chromosome: *PSCA*, *LY6K*, *SLURP1*, *LYPD2*, *LYNX1/SLURP2*, *LY6D*, *GML*, *LY6E*, *LY6L*, *LY6H*, and *GPIHBP1*) (http://genome-euro.ucsc.edu). The syntenic region on mouse Chromosome 15 (15D3-15E1) contains *Psca*, *Slurp1*, *Lypd2*, *Slurp2*, *Lynx1*, *Ly6d*, *Ly6g6g*, *Ly6k*, *Gml*, *Gml2*, *Ly6m*, *Ly6e*, *Ly6i*, *Ly6a*, *Ly6c1*, *Ly6c2, Ly6a2*, *Ly6g*, *Ly6g2*, *Ly6f*, *Ly6l*, *Ly6h*, and *Gpihbp1.* Other smaller clusters are seen on human chromosome 19 (19q13) (*LYPD4*, *CD177*, *TEX101*, *LYPD3*, *PINLYP*, *PLAUR*, *LYPD5*, and *SPACA4* with syntenic region on mouse Chromosome 7 containing *Lypd5*, *Plaur*, *Pinlyp*, *Lypd3*, *Tex101*, *Lypd10*, *Lypd11*, *Cd177*, *Lypd4*, and *Spaca4*), human chromosome 11 (11q24.2) (*ACRV1*, *PATE1*, *PATE2*, *PATE3*, and *PATE4* with syntenic region on mouse Chromosome 9 containing *Pate4*, *Pate2*, *Pate13*, *Pate3*, *Pate1*, *Pate10*, *Pate7*, *Pate6*, *Pate5*, *Pate12*, *Pate11*, *Pate9*, *Pate8*, *Pate14*, and *Acrv1*), and human chromosome 6 (6p21) (*LY6G6C*, *LY6G6D*, *LY6G6F*, *LY6G5C*, and *LY6G5B* with syntenic region in the MHC class III region of the mouse Chromosome 17 containing *Ly6g6c*, *Ly6g5c*, *Ly6g5b*, *Ly6g6d*, *Ly6g6f*, and *Ly6g6e*), while the remaining family members are found on other chromosomes (Tables 1 and 2).

## Typical Ly6/uPAR gene structure

**Fig. 1:**
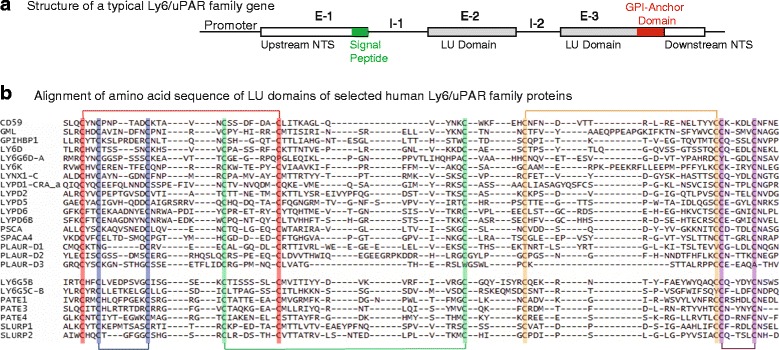
Structure of a typical Ly6/UPAR family gene and alignment of amino acid sequences of selected LU domains. **a** Structure of a typical LY6/UPAR family gene, showing three exons (E-1, E-2 and E-3), two introns (I-1 and I-2), and the location of signal peptide as well as GPI-anchor domain. **b** Alignment of LU domain amino acid sequences of selected human LY6/UPAR proteins. GPI-anchored (*top*) and secreted (*bottom*) proteins are clustered together, with an empty line in between. Alignments were performed using ProbCons in Bioinformatics toolkit provided by Max-Planck Institute for Developmental Biology (http://toolkit.tuebingen.mpg.de). Conserved cysteines linked by non-variant disulfide bridges are highlighted in similar colors. Isoforms are denoted with a *dash* and the isoform name (e.g. LY6G6D-A). LYNX1-C and SLURP2 are precursor forms of the final protein. NTS, Non-translated sequence

Ly6/uPAR family members typically contain one LU domain, with the exception of *LYPD3* [7] and *CD177* [8] which contain two, and *PLAUR* [9], which contains three direct repeats of the LU domain (Tables 1 and 2). The mouse *Cd177* differs from its human ortholog in that it contains four direct repeats of the LU domain. A typical Ly6/uPAR family gene consists of three exons and two introns (Fig. 1a), with the signal peptide being encoded in the first exon. The mature polypeptide is encoded by the last two exons, with the GPI-anchor domain encoded by the third exon.

Based on their subcellular localization, Ly6/uPAR family members are further subdivided into two groups: membrane-tethered (through a GPI-anchor domain) or secreted (lacking the GPI-anchor domain). GPI-anchored Ly6/uPAR family members tend to congregate on lipid rafts on the cell surface, which promotes their interactions with other proteins. A fraction of the GPI-anchored Ly6/uPAR family proteins such as PLAUR are secreted after their GPI-anchor domain is proteolytically cleaved [10–12]. Experimental evidence supports the presence of a GPI-anchoring signal peptide in a majority of Ly6/uPAR family members, while it is absent in a few (Table 3). For those with no experimental evidence, the GPI-anchor signal predictor ‘PredGPI’ program (http://gpcr.biocomp.unibo.it/predgpi/) [13] predicted the presence of a GPI-anchor signal within mouse and human LYPD8 and LY6L, and in mouse LYPD10, LYPD11, LYPD9, LY6F, and LY6M, while predicting its absence in mouse and human LYPD4, LY6G6F, and PINLYP, and in mouse GML2 and LY6G6 (Table 3).

**Table 3:** Expression patterns, interacting factors and cellular functions of mouse Ly6/uPAR family proteins

Approved protein symbol	Human ortholog?	Expression pattern	Interacting factors	Cellular function	Gene knockout?	GPI-anchor?	References
ACRV1	Yes	Spermatazoa (acrosomes)	TARDBP (TDP-43)	Sperm-oolemma binding/penetration	No	No	[29, 31, 109–111]
CD177	Yes	Neutrophils	PR3, PECAM1	Neutrophil activation	Yes	Yes	[46, 47, 112–114]
CD59A	No	Neurons, T cells, splenic macrophages, spermatids	MAC Complex	Complement inhibitor	Yes	Yes	[115–118]
CD59B	Yes (as CD59)	Lymphocytes, granulocytes, platelets, erythrocytes, activated T cells, spermatazoa, splenic macrophages, mature spermatozoa	C8, C9	Complement inhibition, cell-cell adhesion	Yes	Yes	[17, 48, 56, 67, 118–120]
GML	Yes	Cancer cells	TRP53	Apoptosis	No	Yes	[121, 122]
GML2	No	Hematopoetic stem cells	Unknown	Unknown	No	Predicted No	[123]
GPIHBP1	Yes	Capillary endothelial cells	LPL	Chylomicron processing, LPL transport	Yes	Yes	[62, 63, 124]
LY6A	No	Hematopoetic stem cells, B cells, T cells, DCs	IFN-γ, MMPs	T cell activation and proliferation controller	Yes	Yes	[28, 125–129]
LY6A2	No	Unknown	Unknown	Unknown	No	Predicted Yes	
LY6C1	No	T cells, NK cells, monocytes, neutrophils, DCs, bone marrow myeloid cells	Type I and II interferons, LFA-1	CD8^+^ T cell migration	No	Yes	[28]
LY6C2	No	T cells, NK cells, monocytes, neutrophils, DCs, bone marrow myeloid cells	Type I and II interferons, LFA-1	CD8^+^ T cell migration	No	Yes	[28]
LY6D	Yes	Keratinocytes, B cells	Camptothecin, mitomycin C, carboplatin, hydroxyurea, aphidicolin	Cell adhesion, B cell specification	No	Yes	[130–134]
LY6E	Yes	Myeloid cells, thymocytes	Retinoic acid, INF1	Monocyte inhibitor, T cell development	No	Yes	[135–139]
LY6F	No	Non-lymphoid tissues	Unknown	Unknown	No	Predicted Yes	[140]
LY6G	No	Neutrophils, granulocytes	LTB_4_, β2-integrins	Neutrophil recruitment	No	Yes	[28, 109, 141]
LY6G2	No	Unknown	Unknown	Unknown	No	Predicted No	
LY6G5B	Yes	Stomach, placenta	Unknown	Unknown	No	No	[142, 143]
LY6G5C	Yes	Testis, fetal liver/spleen/retina/heart/thymus, MS lesions, germinal center B cells, carcinoid lung	Unknown	Hematopoietic cell differentiation	No	No	[142, 143]
LY6G6C	Yes	Fibroblasts	Unknown	Filipodia functions	No	Yes	[142, 143]
LY6G6D	Yes	Fibroblasts	Unknown	Filipodia functions	No	Yes	[142, 143]
LY6G6E	No	Undifferentiated megakaryocyte-like cells	Unknown	α_4_β_2_ potentiator, cell adhesion and migration	No	Yes	[105, 142]
LY6G6F	Yes	K562 acute myeloblastic leukemia cells	GRB2, GRB7	Unknown	No	Predicted No	[144]
LY6G6G	No	Unknown	Unknown	Unknown	No	Predicted No	
LY6H	Yes	Exocrine and endocrine epithelial cells, CNS	Unknown	Unknown	No	Yes	[145, 146]
LY6I	No	Monocytes, granulocytes, CD19^+^ B cells, thymocytes and T cells	Unknown	Unknown	No	Yes	[28, 147]
LY6K	Yes	Keratinocytes, squamous cell carcinomas	JUND, FOSL1, RAS	Invasive cell metastasis	Yes	Yes	[148–152]
LY6L	Yes	Unknown	Unknown	Unknown	No	Predicted Yes	
LY6M	No	Unknown	Unknown	Unknown	No	Predicted Yes	
LYNX1	Yes	CNS neurons	α_4_β_2_ and α_7_ nAChRs	nAChR modulator	Yes	Yes	[25, 66, 73, 153]
LYPD1	Yes	Airway epithelial cells, embryonic tissue, CNS and PNS neurons	α_4_β_2_, α_4_β_4_, and α_7_ nAChRs	α7 nAChR modulator, tumor suppression	Yes	Yes	[103, 104, 154, 155]
LYPD2	Yes	Unknown	α_4_β_2_ nAChRs	nAChR Modulator	No	Yes	[105]
LYPD3	Yes	Stratum spinosum keratinocytes, cancer cells	α_6_β_4_, MMP14	Metastasis, EMT, wound healing	No	Yes	[156–160]
LYPD4	Yes	Unknown	Unknown	Unknown	No	Predicted No	
LYPD5	Yes	Stratum granulosum keratinocytes	AP-1	Unknown	No	Yes	[156, 161]
LYPD6	Yes	Ubiquitously	WNT3A	Tumor suppression	No	Yes	[162–164]
LYPD6B	Yes	Testis, lung, stomach, prostate	AP-1	PKC signal transduction pathway	No	Yes	[165]
LYPD8	Yes	Unknown	Unknown	Unknown	No	Predicted Yes	
LYPD9	No	Unknown	Unknown	Unknown	No	Predicted Yes	
LYPD10	No	Unknown	Unknown	Unknown	No	Predicted Yes	
LYPD11	No	Unknown	Unknown	Unknown	No	Predicted Yes	
PATE1	Yes	Leydig cells, testicular germ cells, prostatic epithelial cells principle cells, spermatazoa,	INCA1	Spermatazoa/egg fusion and penetration, spermatazoa motility	No	No	[29, 32, 166–168]
PATE2	Yes	Spermatazoa, testicular germ cells, neuronal tissue	nAChR	nAChR modulator	No	No	[29, 31]
PATE3	Yes	Testis	nAChR	nAChR modulator	No	No	[29]
PATE4	Yes	Spermatazoa, prostatic apical epithelial cells, spinal cord tissue	α_7_ nAChR	nAChR modulator	Yes	No	[29, 33]
PATE5	No	Male reproductive organs	Androgens, lumicrine testicular factors	Unknown	No	No	[29, 30]
PATE6	No	Male reproductive organs	Androgens	nAChR regulator	No	No	[29, 30]
PATE7	No	Male reproductive organs	Androgens	Unknown	No	No	[29, 30]
PATE8	No	Male reproductive organs, skeletal muscle	Androgens	Unknown	No	No	[29, 30]
PATE9	No	Male reproductive organs	Unknown	Unknown	No	No	[29, 30]
PATE10	No	Male reproductive organs	Androgens, lumicrine testicular factors	Unknown	No	No	[29, 30]
PATE11	No	Placenta	α4β2 nAChR	α_4_β_2_ nAChR regulator	No	No	[29, 30]
PATE12	No	Placenta	Unknown	Unknown	No	No	[29, 30]
PATE13	No	Male reproductive organs	Androgens	Unknown	No	No	[29]
PATE14	No	Luminal epithelium of seminal vesicles	Androgens	Unknown	No	No	[169]
PINLYP	Yes	Unknown	Unknown	Unknown	No	Predicted No	
PLAUR	Yes	Airway epithelial cells, trophoblast cells, cancerous cells, HUVEC, pan-epithelial	uPA, Mac-1, caveolin, SERPINE1, vitronectin, kininogen, thrombospondin, α2-macroglobulin receptor, MRC2, cation-independent mannose 6-phosphate/insulin-like growth factor II receptor	Fibrinolysis, matrix remodeling, cell migration, growth factor activation, integrin regulation, tumor cell invasion, cell adhesion	Yes	Yes	[23, 170–185]
PSCA	Yes	Prostate basal cells, epithelial cells of prostate, urinary bladder, kidney, skin, esophagus, stomach, and placenta, cortical cells	SNAI2, α4 nAChR	Tumor suppression, oncogene, nAChR modulator	Yes	Yes	[26, 57–59, 61, 150, 186–188]
SLURP1	Yes	Keratinocytes, mucocutaneous and aerodigestive epithelia, some C-fiber neurons	α_7_ nAChR, KLF4	Tumor suppression, nAChR modulator	Yes	No	[20, 76, 79, 94, 99, 189–192]
SLURP2	Yes	Oral and epidermal keratinocytes	α_3_ nAChR	nAChR modulator, blocks apoptosis	Yes	No	[99–102, 193]
SPACA4	Yes	Spermatazoa	Unknown	Spermatazoa-egg binding/fusion	No	Yes	[194]
TEX101	Yes	Spermatazoa, cancerous tissues	Progesterone, PLAU	Spermatazoaatogenesis, spermatazoa-egg interaction, protease suppressor	Yes	Yes	[151, 195–198]

## Structure of the LU domain

The Ly6/uPAR family members have a well-conserved LU domain with a characteristic three-finger structure formed by disulfide bridges connecting the conserved cysteine residues in a specific pattern. LU domains are topologically similar to the three-finger structure of snake venom neurotoxins, which have three β-sheet loops fixed in space by virtue of their unique disulfide bridges. The structure of the extracellular region of CD59 was first solved by 2D NMR methods [14, 15] and further refined by crystallography [16] revealing it to be a flat, disk-shaped molecule consisting of a two-stranded beta-sheet finger loosely packed against a protein core formed by a three-stranded beta-sheet and a short helix.

Alignment of LU domain amino acid sequences of selected human LY6/UPAR proteins performed using ProbCons (http://toolkit.tuebingen.mpg.de) revealed the location of conserved cysteines (Fig. 1b). Five well-conserved disulfide bridges between cysteine pairs 3 and 26, 6 and 13, 19 and 39, 45 and 63, and 64 and 69 stabilize the hydrophobic core, from which three β-sheet-based fingers protrude (Fig. 1b). The sequence of the amino acids exposed at the tips of each finger as well as the length of each of the fingers is variable, providing the three-finger motif with the flexibility for a wide range of intermolecular interactions. In addition to the LU domain, Ly6/uPAR family proteins possess a well-conserved LeuXxxCysXxxXxxCys motif at the amino-terminus and CysCysXxxXxxXxxXxxCysAsn motif at the carboxyl-terminus (Fig. 1b). Functional relevance of these motifs is not yet known.

Most Ly6/uPAR family proteins maintain the ten cysteines characteristic of the LU domain, with some notable exceptions. In PLAUR, which consists of three LU domains (designated D1, D2 and D3), only domain D2 is fully intact with ten cysteines, while domains D1 and D3 have seven and eight cysteines, respectively. Isoforms of proteins such as human LY6G5C maintain conservation throughout the LU domain in almost every isoform. In contrast, different isoforms of human LYNX1 maintain the necessary cysteines, but little else is conserved (Fig. 1b).

## Expression of Ly6/uPAR family genes

The expression pattern, interacting factors, and cellular functions of the mouse and human Ly6/uPAR family members are summarized in Table 3. Expression of Ly6/uPAR proteins is (i) widespread and variable across diverse cell types and tissues, (ii) tightly regulated in a spatiotemporal manner, and (iii) often correlated with cellular differentiation. Although the Ly6/uPAR family protein structures are well-conserved across species, their expression patterns tend to vary, indicating divergence among their regulatory networks. Many Ly6/uPAR family members are expressed in hematopoietic precursors in a lineage-specific fashion making them useful cell surface markers for leukocytes, facilitating identification of individual leukocyte subgroups [17–19]. For example, mouse myeloid differentiation marker LY6G (also called Gr-1) is expressed by the myeloid lineage cells in a developmentally regulated manner in the bone marrow. Anti-LY6G antibodies are routinely used to identify neutrophils in the mouse but not humans as there is no human ortholog for *Ly6g*. Ly6/uPAR family members are generally upregulated during inflammatory conditions or infections and in cancerous cells, with a notable exception of SLURP1, which is invariably downregulated in pro-inflammatory conditions [9, 20–24].

## Functions of Ly6/uPAR family proteins

Commensurate with their varied expression patterns, Ly6/uPAR proteins have a wide range of functions in cell proliferation, migration, cell-cell interaction, immune cell maturation, macrophage activation, and cytokine production. They typically exert their influence by targeting nicotinic acetylcholine receptors (nAChRs) (reviewed in [1]). GPI-anchored Ly6/uPAR proteins lacking a cytoplasmic tail are unable to directly participate in intracellular signaling but can initiate signaling by interacting with other transmembrane proteins. Such interactions of GPI-anchored proteins are further facilitated by their tendency to congregate in lipid rafts on the cell surface, where other signaling molecules also are enriched. While GPI-anchored Ly6/uPAR proteins control signaling through interaction with their ligand(s), secreted Ly6/uPAR proteins may serve as agonists for other receptors including nAChR and/or competing scavengers of their ligands [1, 20, 21, 25–27]. Many Ly6/uPAR family members have a prominent role in neutrophils (Table 3) [28]. Below, we summarize the functions of a few well-studied members.

## Prostate and testis expressed genes

Human chromosome 11 contains 5 prostate and testis expressed (PATE) genes while the syntenic region on murine Chromosome 9 contains 15 genes [29]. Recent evidence demonstrates that PATE proteins are much more predominantly expressed in the epididymis with a significantly lower expression in the prostate and testis, suggesting that their names are misnomers [30]. PATE proteins secreted by epithelial cells to the epididymal lumen facilitate spermatozoan maturation as they leave the testis and travel through the epididymis. PATE proteins localized in the sperm head assist in sperm-oolemma fusion and penetration [31]. Defects in PATE1 result in decreased sperm motility in aged men and young asthenozoospermia patients, revealing the molecular basis for the decline in sperm quality with age [32]. PATE4 is abundantly expressed in the mouse prostate, spermatozoa, and seminal vesicles. *Pate4*−/− mice remain fertile and do not display any histological abnormalities [33]. PATE proteins are also expressed in neuron-rich tissues, where they function by modulating nAChR activities [29].

## Plasminogen activator, urokinase receptor

Also known as the urokinase-type plasminogen activator receptor (uPAR), plasminogen activator, urokinase receptor (PLAUR) is the most well-studied family member [9]. It is widely expressed in different cell types and plays a key regulatory role in cell surface plasminogen activation, influencing many normal and pathologic processes [9, 23]. PLAUR consists of three direct repeats of the LU domain, which together bind urokinase-type plasminogen activator (PLAU/uPA) in both the pro-protein and mature forms. PLAUR (i) expression is regulated by KLF4 [34] and is upregulated in cancer cells [35, 36] and in response to pro-inflammatory conditions [37], (ii) facilitates neutrophil recruitment in response to bacterial infection [38], (iii) facilitates clearance of Borrelia infection [39], and (iv) interacts with multiple partners including vitronectin and different integrins. Although the bulk of PLAUR exists as GPI-anchored, some of it is known to be secreted as “soluble uPAR” (suPAR), the expression level of which is correlated with disease conditions [10–12, 40].

PLAUR is a multi-functional protein with important roles in regulating cell-matrix interaction, motility, and immune response. PLAUR expression levels directly correlate with the invasive potential of endometrial carcinomas, suggesting that it is a valuable prognostic marker for aggressive endometrial tumors [35]. PLAUR expression is normally low in healthy glomeruli and is elevated in glomeruli from individuals with focal segmental glomerulosclerosis, consistent with its role in regulating renal permeability [41]. PLAUR is required for neutrophil recruitment into alveoli and lungs in response to *S. pneumoniae* infection [42]. *Plaur*−/− macrophages display an enhanced ability to engulf wild-type neutrophils, but *Plaur*−/− neutrophils do not, suggesting that PLAUR plays an essential role in recognition and clearance of neutrophils [43]. *Plaur*−/− mice exhibit abnormal interneuron migration from the ganglionic eminence, and reduced interneurons in the frontal and parietal cortex [44, 45].

## CD177

Expressed by neutrophils, neutrophilic metamyelocytes, and myelocytes, CD177 mediates neutrophil migration across the endothelium by binding PECAM1 (CD31). Anti-CD177 antibodies inhibit neutrophil transmigration across the endothelial monolayer, potentially by interfering with an interaction between CD177 and PECAM1 [46]. Mutations in *CD177* or its dysregulated expression are associated with myeloproliferative diseases, secondary to a gain-of-function mutation in *JAK2* [8]. Exposure of human neutrophils to pulmonary endotoxin results in strong upregulation of *CD177* [47]. Expression of *CD177* mRNA is highly upregulated following endotoxin exposure. Overexpression of CD177 is a biomarker for thrombocythemia patients with elevated risk of thromboembolic complications [8]. While human *CD177* contains nine exons that encode a protein with two LU domain repeats, mouse *Cd177* is substantially larger with 17 exons that encode a larger protein with four LU domain repeats. Surprisingly, *Cd177*−/− mice displayed no discernible phenotype or any change in immune cells, other than decreased neutrophil counts in peripheral blood [47]. Absence of CD177 had no significant impact on CXCL1/KC- or fMLP-induced mouse neutrophil migration, but led to significant cell death [47].

## Complement regulatory protein CD59

CD59 is an essential regulatory protein that protects hematopoietic and neuronal cells against complement-mediated osmolytic pore formation by binding C8 and/or C9 and inhibiting the incorporation of C9 into the membrane attack complex [17, 48–51]. CD2-mediated CD59 stimulation results in secretion of IL1A (IL-1α), IL6, and CSF2 (GM-CSF) in keratinocytes [52]. Inadequate complement regulation is associated with age-related macular degeneration [53]. Mutations in *CD59* cause uncontrolled complement activation in hemolytic anemia, thrombosis, and cerebral infarction in paroxysmal nocturnal hemoglobinuria [54]. The mouse genome contains two homologs of *CD59*, termed *Cd59a*, and *Cd59b*. Mouse CD59B has approximately a sixfold higher specific activity than CD59A and is considered a true ortholog of human CD59. *Cd59a* deficiency exacerbated the skin disease and lymphoproliferative characteristic of the MRL/lpr murine lupus model suggesting that CD59A inhibits systemic autoimmunity in the MRL/lpr lupus model through a complement-independent mechanism [55]. Consistent with its higher specific activity, *Cd59b*−/− mice display a stronger phenotype including hemolytic anemia, anisopoikilocytosis, echinocytosis, schistocytosis, hemoglobinuria with hemosiderinuria, and platelet activation [56]. *Cd59b*−/− males suffer from progressive loss of fertility after 5 months of age [56].

## Prostate stem cell antigen

Prostate stem cell antigen (PSCA) is a 123 amino acid protein with an N-terminal signal sequence, and a C-terminal GPI-anchoring sequence [57]. It was initially identified as a prostate-specific cell surface antigen in normal male tissues and found to be highly expressed in human prostate cancer [58, 59]. Later studies have revealed it to be more widely expressed. A genome-wide association study of Japanese patients with gastric cancer revealed that genetic variation in *PSCA* is associated with susceptibility to diffuse-type gastric cancer [60]. *Psca*−/− mice are viable, and fertile, with similar rates of spontaneous or radiation-induced primary epithelial tumor formation as the wild-type mice. However, *Psca*−/− mice display an increased frequency of metastasis suggesting that PSCA may play a role in limiting tumor progression, and deletion of *Psca* promotes tumor migration and metastasis [61].

## GPI-anchored high density lipoprotein-binding protein 1

GPI-anchored high density lipoprotein-binding protein 1 (GPIHBP1) is an endothelial cell protein expressed on the luminal face of capillaries in brown adipose tissue, heart, lung, and liver. GPIHBP1 binds high density lipoprotein and provides a platform for lipoprotein lipase (LPL)-mediated processing of chylomicron lipoprotein particles which transport dietary lipids from the intestines to other locations in the body. GPIHBP1 mutations that affect its ability to bind LPL or chylomicrons are associated with chylomicronemia [62–64]. *Gpihbp1*−/− mice cannot transport lipoprotein lipase to the capillary lumen, resulting in mislocalization of lipoprotein lipase within tissues, defective lipolysis of triglyceride-rich lipoproteins, and chylomicronemia [62–64]. Defective lipolysis causes reciprocal metabolic perturbations in *Gpihbp1*−/− mouse adipose tissue and liver. The essential fatty acid content of triglycerides is decreased and lipid biosynthetic gene expression is increased in adipose tissue, while the opposite changes occur in the liver [65].

## Ly6/neurotoxin-1

As an allosteric modulator of nAChR function, Ly6/neurotoxin-1 (LYNX1) serves as a cholinergic brake that limits neuronal plasticity, balancing neuronal activity, and survival in the adult visual cortex [25, 66–68]. LYNX1 also inhibits SRC activation, suppressing mucin expression in the airway epithelium [69]. The *LYNX1* gene is positioned in close proximity to *SLURP2*, leading to the mistaken idea that they are alternatively spliced isoforms of the same gene, a theory which was disproved recently [70]. *LYNX1* is one of the genes that has shown accelerated evolution in humans relative to other primates, correlating with the increased brain size and complexity [71]. The juvenile brain exhibits high plasticity which is severely restricted in adulthood. Adult *Lynx1*−/− mice exhibited visual cortex plasticity similar to that of juveniles, suggesting that LYNX1 serves as a break for cortical plasticity [68]. Using the mouse model, it was demonstrated that LYNX1 plays a modulatory role in the aging brain, and that soluble LYNX1 may be useful for adjusting cholinergic-dependent plasticity and learning mechanisms [72–74].

## Secreted Ly6/urokinase-type plasminogen activator receptor-related protein 1

Secreted Ly6/urokinase-type plasminogen activator receptor-related protein 1 (SLURP1) is expressed in a variety of cells including immune cells [75], sensory neurons [76], and epithelial cells [77–80], and secreted into plasma, saliva, sweat, urine, and tears [22, 81]. *SLURP1* is downregulated in corneal neovascularization [82], asthmatic lungs [83], Barrett’s esophagus [84, 85], malignant melanomas [86], and squamous cell carcinomas [87, 88]. Mutations or deletions in *SLURP1* cause autosomal recessive palmoplantar hyperkeratotic disorder ‘mal de Meleda’ [78, 81, 89–93]. SLURP1 is structurally similar to the snake and frog cytotoxin α-bungarotoxin, and acts as a CHRNA7 (α7nAChR)-ligand, regulating keratinocytes through cholinergic pathways [78, 94]. It modulates signal transduction, activation of the immune response, and cell adhesion, and blocks malignant transformation [75, 79, 95–97]. SLURP1 is proposed to modulate acetylcholine signaling through CHRNA7 [98]. We have documented that SLURP1 serves as an important immunomodulatory molecule at the ocular surface by acting as a soluble scavenger of urokinase (PLAU) [20–22]. *Slurp1−/−* mice develop signs of palmoplantar keratoderma including elevated keratinocyte proliferation, accumulation of lipid droplets in the stratum corneum, and defective epidermal barrier function reminiscent of mal de Meleda. *Slurp1−/−* mice also display decreased adiposity, low plasma lipid levels, and a neuromuscular abnormality (hind-limb clasping), suggesting additional functions for SLURP1 [99].

## Secreted Ly6/urokinase-type plasminogen activator receptor-related protein 2 (SLURP2)

SLURP2 is expressed by human epidermal and oral keratinocytes, from where it is secreted into sweat and saliva, respectively [100]. *SLURP2* expression is strongly induced in psoriatic skin lesions possibly by IL22, and is blocked by IFNG [70, 101]. SLURP2 blocks the effect of acetylcholine by binding CHRNA3 (α3nAChR), and delays keratinocyte differentiation and prevents apoptosis [100]. Although the *SLURP2* and *LYNX1* genes are closely linked leading to a mistaken idea that they are isoforms, it is now clear that they constitute separate transcription units that are differently regulated [70]. *Slurp2−/−* mice also develop signs of palmoplantar keratoderma and neuromuscular abnormality (hind-limb clasping) reminiscent of those seen in *Slurp1−/−* mice [99, 102].

## Ly6/Plaur domain containing 1

Ly6/Plaur domain containing 1 (LYPD1), also known as LYNX2, is a prototoxin gene that is expressed in postmitotic central and peripheral neurons including subpopulations of motor neurons, sensory neurons, interneurons, and neurons of the autonomous nervous system [103]. LYPD1 is expressed at high levels in anxiety associated brain areas and plays an important role in regulating anxiety by binding and modulating neuronal nicotinic receptors [104, 105]. Ablation of *Lypd1* alters the actions of nicotine on glutamatergic signaling in the prefrontal cortex, resulting in elevated anxiety-like behaviors [104].

## Evolution of Ly6/uPAR family proteins

Ly6/uPAR family genes are conserved across species suggesting that they are evolutionarily ancient. Organization of the genes in this family in clusters on multiple chromosomes suggests that both gene duplications and translocations have played a role in their evolution. Comparison of the mouse and human Ly6/uPAR family genes reveals that while there are many orthologs, some Ly6 genes are only present in the mouse. The Ly6 gene complexes on human chromosomes 8, 19, 11, and 6 are syntenic with their counterparts on mouse Chromosomes 15, 7, 9, and 17, respectively, suggesting that these gene complexes were already present in their common ancestor. There are no human orthologs for the subcluster of murine Ly6 genes *Ly6i*, *Ly6a*, *Ly6c1*, *Ly6c2*, *Ly6a2*, *Ly6g*, *Ly6g2*, and *Ly6f* on Chromosome 15, and *Pate10*, *Pate7*, *Pate6*, *Pate5*, *Pate12*, *Pate11*, *Pate9*, *Pate8*, and *Pate14* on Chromosome 9, suggesting that these regions may have arisen in the mouse through gene duplication after evolutionary divergence of these two species. What their functions are in the murine neutrophils and epididymis, respectively, where they are abundantly expressed, and how they are compensated in the corresponding human tissues, remains to be determined.

**Fig. 2:**
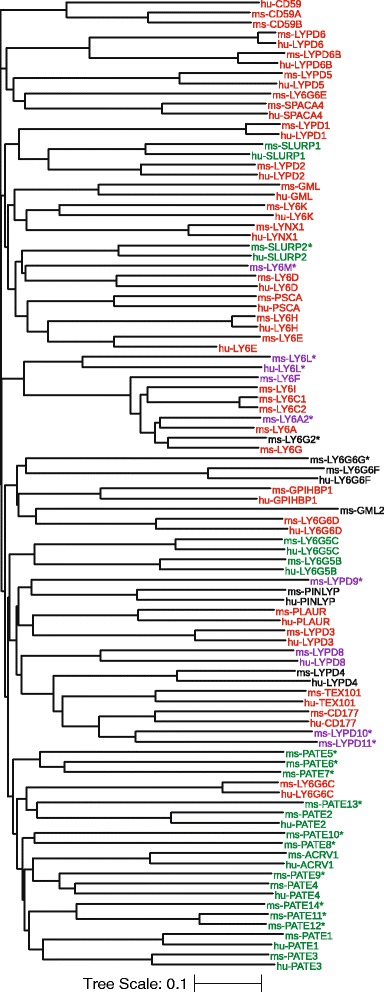
Phylogram revealing the evolutionary relationship among mouse (ms-) and human (hu-) Ly6/uPAR family proteins. The phylogram was generated using the amino acid sequences in Clustal-Omega web-based program [106, 107] (http://www.ebi.ac.uk/Tools/msa/clustalo/). The display was generated using the methods described [108]. The length of each branch from the most recent branch point indicates the evolutionary distance, or the relative period of time the protein has been in its current state. Known GPI-anchored Ly6/uPAR family proteins are shown in *red*, and those secreted (without GPI-anchor) are shown in *green*. Those predicted to contain a GPI-anchor (but not yet experimentally proven) are in *purple*, and those predicted to not contain a GPI-anchor sequence (but not yet experimentally proven) are in *black*. Novel genes named in this study are indicated with an asterisk (*)

In order to evaluate the evolutionary relatedness of LU family proteins, we generated a phylogram by multiple sequence alignment of their amino acid sequences using web-based Clustal-Omega software, and visualized it with web-based software from Interactive Tree of Life (Fig. 2) [106–108]. Where multiple isoforms exist, we only used the sequence of the longest isoform. Analysis of the phylogenetic relationship among human and mouse Ly6/uPAR family proteins revealed that (i) human LY6K and mouse GML2 are the most ancestral Ly6 proteins with the longest unbranched streak in these two species, (ii) human and mouse LYPD6 are the most recent addition to the family closely followed by mouse LY6C1 and LY6C2, (iii) most of the secreted family proteins (with the notable exception of SLURP1 and SLURP2) form a separate cluster distinct from the GPI-anchored proteins, and (iv) several mouse PATE proteins (PATE4, 5, 6, 7, 8, 9, 10, 13, and 14) have long unbranched streaks suggesting that they have ancient origin and that the important function(s) that they serve have not changed much (Fig. 2).

## Concluding remarks

In this gene family update, we have summarized the current literature on the organization, expression patterns, functions, and evolution of human and mouse Ly6/uPLAR family genes. In addition, we identified and named many novel Ly6/uPAR family members. Considering that Ly6/uPLAR family members play critical roles in regulating immunological and physiological responses to infections and varying environmental conditions, it is imperative that we understand them in greater detail. Their involvement in regulating a wide range of functions such as progression of inflammation, complement activity, neuronal activity, angiogenesis, wound healing, and cancer growth indicates that Ly6/uPAR family members will be useful therapeutic targets. Additional insight into (i) the biological functions of individual family proteins, (ii) signaling cascades that regulate their expression and functions, and (iii) the identity of their interacting partners is expected to herald new modalities for diagnosis and treatment of diverse diseases.
